# Supplementary material for: Modelling the drivers of the spread of Plasmodium falciparum hrp2 gene deletions in sub-Saharan Africa
Source: eLife. 2017 Aug 24;6:e25008. doi: 10.7554/eLife.25008 (PMC5602420; doi:10.7554/eLife.25008)
Supplement: Supplementary file 1: — Mathematical style pseudocode description of the simulation model. [file elife-25008-supp1.docx]

**Supplementary File 1: Simulation Model Pseudocode**

The following key and colour key describe the pseudocode convention that is used in the following pseudocode representation of the full model. A mathematical style pseudocode syntax style has been used to aid understanding and reproducibility of the model.

**KEY:**

| **Symbol/Language** | **Type of Operation** |
| --- | --- |
| := | Assignment |
| = > < <= >= | Comparison |
| + - * / ^ | Arithmetic |
| AND OR | Logical |
| x FROM y EARLIER | Value of x from y days earlier |
| DRAW FROM [x_1_,...,x_n_] WITH WEIGHTS [w_1_,...,w_n_]  DRAW UNIFORMLY FROM [x1,...,xn] | Randomly sample value from vector of values x_1_,...,x_n_ with probability of value being sampled equal to [w_1_,...,w_n_]  Randomly sample value from vector vector of values x1,...,xn |
| REPEAT LINES l_1_-l_2_ | Repeat code between and including lines l_1 ­_and l_2_ |
| FOR TO ENDFOR  IF ELSE ENDIF  WHILE ENDWHILE | Control of pseudocode flow |

**COLOUR KEY:**

| **Colour** | **Explanation** |
| --- | --- |
| 001 | Line numbers in blue at beginning of pseudocode lines |
| // Annotation | Pseudocode annotation and explanations in green |
| OPERATORS, SYMBOLS, KEYWORDS | Mathematical operators, and instructions detailed in the above key are in red capitals |
| Variables and functions | Variables and values are in black lowercase, as well as intelligible functions [round(), mean()], sampling randomly from distributions [randexp(), randlognormal()] and carrying out bernoulli trials [bernoulli()]. Variable symbols relate to the model parameters within Table 2 in the main text. |

// BEGIN HUMAN INITIALISATION

// ----------------------------------------------------

001 FOR i := 1 TO N // N is total population size

//assign age

002 age[i] := randexp($\mu$) // set individual's age to random draw from exponential distribution with mean equal to 1/$\mu$ years

003 age[i] := round(age[i]) // round age to nearest integer

004 WHILE (age[i] > (100*365)) // reassign age if greater than max age of 100 years

005 REPEAT LINES 02-03

006 ENDWHILE

// assign relative biting rate due to heterogeneity in mosquito biting patterns

007 $\zeta$[i] := randlognormal(meanlog:=-0.5*$\sigma^{2}$,sdlog:=$\sqrt{\sigma^{2}}$) // set individual’s relative biting rate to random draw from lognormal distribution with mean and standard deviation of the distribution on the log scale equal to -0.5*$\sigma^{2}$ and $\sqrt{\sigma^{2}}$ respectively.

008 inf_state[i] := DRAW FROM [S,D,A,U,T,P] WITH WEIGHTS [S_eq,_D_eq,_A_eq,_U_eq,_T_eq,_P_eq_] // S_eq_ is the steady state equilibrium proportion of individuals who are susceptible within a near-equivalent deterministic model^1^ for the desired EIR, and so on for D,A,U,T,P

// assign strain profile, i.e. number of wild type of hrp2-deleted strains

009 IF (inf_state[i] = D OR A OR U OR T) //if individual is infected

// first assign what strains they have

010 n_wt_strains[i] := DRAW UNIFORMLY FROM [0,...,5] // assign a random number of wild type strains from between 0 and 5

011 n_hrp2_strains[i] := DRAW FROM [0,...,5] WITH WEIGHTS [hrp2_freq_wt_0,...,hrp2_freq_wt_5] // hrp2_freq_wt weights are such that the population allele frequency of hrp2 deleted strains is the desired starting frequency.

// reassign if infected individual did not have any strains assigned

012 WHILE (n_wt_strains[i] + n_hrp2_strains[i] = 0)

013 REPEAT LINES 10-11

014 ENDWHILE

015 ENDIF

// assign immunity using equilibrium state

016 IB[i] := IB_eq_[age[i],$\zeta$[i]] // assign pre-erythrocytic immunity from steady state equilibrium immunity for the age and biting heterogeneity class that individual i exists in

017 ICA[i] := ICA_eq_[age[i],$\zeta$[i]] // assign acquired clinical immunity from steady state equilibrium immunity for the age and biting heterogeneity class that individual i exists in

018 ID[i] := ID_eq_[age[i],$\zeta$[i]] // assign detection immunity from steady state equilibrium immunity for the age and biting heterogeneity class that individual i exists in

019 ICM[i] := P_M_*ICA_eq_[20*365]*exp(-age[i]*r_CM_^­^) // assign maternal immunity using the mean equilibrium steady state acquired clinical immunity for the age class that contains the assumed maternal age of 20 years, multiplied by relative immunity passed on parameter P_M_ multiplied by how much this immunity would have decayed given the age of the individual.

020 t_k_[i] := t_l_[i] := t_m_[i] := -T_big_ // assign the last time IB, ICA and ID were boosted to a large negative number to represent never having been exposed or infected before.

// assign contributions to onward infectiousness

021 IF (inf_state = A) //if they are asymptomatic

022 f_D_ := 1 - ((1 - f_D0_)/(1 + ((age[i]/a_D_)^$\gamma$_D_)))

023 q := d_1_ + ((1 - d_1_)/(1 + (((ID[i]/I_D0_)^$\kappa$_D_)*f_D_)))

024 cont[i] := c_U_ + (c_D_ - c_U_)*(q^$\gamma$_1_)

025 ELSE // else assign the contribution term for the infection state they are in

026 IF (inf_state[i] = D) // if they are diseased contribution equal to c_D_

027 cont[i] := c_D_

028 ENDIF

029 IF (inf_state[i] = U) // if they are subpatent contribution equal to c_U_

030 cont[i] := c_U_

031 ENDIF

032 IF (inf_state[i] = T) // if they are being treated contribution equal to c_T_

033 cont[i] := c_T_

034 ENDIF

035 ENDIF

// assign non-normalised age dependent biting heterogeneity

036 $\psi$[i] := (1 - $\rho$*exp(-age[i]/a_0_))

037 $\psi$_sum := $\psi$_sum + $\psi$[i]

038 ENDFOR

// normalise age-dependent biting heterogeneity by dividing by the population mean

039 FOR i := 1 TO N

040 $\psi$[i] := $\psi$[i]/($\psi$_sum/N)

041 ENDFOR

// ----------------------------------------------------

// END HUMAN INITIALISATION

// BEGIN MOSQUITO INITIALISATION

// ----------------------------------------------------

042 S_M_ := S_Meq_ // assign susceptible mosquito population size to steady state equilibrium size

043 E_M_ := E_Meq_ // assign exposed mosquito population size to steady state equilibrium size

044 I_M_ := I_Meq_ // assign infectious mosquito population size to steady state equilibrium size

// ----------------------------------------------------

// END MOSQUITO INITIALIATION

// BEGIN STEPPING THROUGH TIME

// ----------------------------------------------------

045 FOR t := 1 TO t_max // t_max is total simulation time in days

// if we are within the first 35 days set the delays equal to 1. This wil cause a slight burn in period, which is accounted for with long initial simulation times to reach equilibrium

046 IF (t < 35)

047 delay_gam := delay_liver := delay_mos := 1

048 ELSE

049 delay_gam := d_g_ // assign delay due to gametocytogenesis

050 delay_liver := d_E_ // assign delay due to liver stage infection

051 delay_mos := d_EM_ // assign delay due to mosquito incubation period

052 ENDIF

055 $\psi$_sum := 0 // reset psi sum to 0

// BEGIN NON-INFECTION CHANGES

// ----------------------------------------------------

056 FOR i := 1 TO N

// update human ages, find individuals who die

// ----------------------------------------------------

057 age[i] := age[i] + 1 // increase age by one day

058 IF (age[i] >= (100*365) OR bernoulli(1-exp(-$\mu$)) = 1) // if older than 100 years or die

059 age[i] := 0 // reset age to 1 day

060 inf_state[i] := S // reset infection state to susceptible

061 n_wt_strains[i] := n_hrp2_strains[i] := 0 // clear all strains

062 IB := ICA := ID := 0 // reset immunities

063 ICM := P_M_*mean(ICA[20*365 < age <= 21*365]) // assign maternal immunity using the mean acquired clinical immunity of individuals who are aged between 20 and 21 years old

064 t_k_[i] := t_l_[i] := t_m_[i] := -T_big_ // reset last biting times

065 $\zeta$[i] := randlognormal(meanlog=-0.5*$\sigma^{2}$,sdlog=$\sqrt{\sigma^{2}}$) //assign biting heterogeneity rate

066 ELSE

// adjust immunities to exponentially decline

067 IB[i] := IB[i]*exp(-r_B_)

068 ICA[i] := ICA[i]*exp(-r_CA_)

069 ID[i] := ID[i]*exp(-r_D_)

070 ICM[i] := ICM[i]*exp(-r_CM_)

071 ENDIF

// Change of infection states not due to an infection

// ----------------------------------------------------

// T -> P

072 IF (inf_state[i] = T) // if they are in state T

073 IF (bernoulli(1-exp(-r_T_)) = 1) // do they recover to state P today

074 inf_state[i] := P // move to state P

075 n_wt_strains[i] := n_hrp2_strains[i] := 0 //clear all strains

076 ENDIF

077 ENDIF

// P -> S

078 IF (inf_state[i] = P) // if they are in state P

079 IF (bernoulli(1-exp(-r_P_)) = 1) // do they lose prophylaxis and become susceptible again today

080 inf_state[i] := S // move to state S

081 ENDIF

082 ENDIF

// D -> A

083 IF (inf_state[i] = D) // if they are in state D

084 IF (bernoulli(1-exp(-r_D_)) = 1) // do they cease having symptoms and move to an asymptomatic infection today

085 inf_state[i] := A // move to state A

086 ENDIF

087 ENDIF

// A -> U

088 IF (inf_state[i] = A) // if they are in state A

089 IF (bernoulli(1-exp(-r_A_)) = 1) // do they cease being detectable as an infection and move to a sub-patent infection today

090 inf_state[i] := U // move to state U

091 ENDIF

092 ENDIF

// U -> S

093 IF (inf_state[i] = U) // if they are in state U

094 IF (bernoulli(1-exp(-r_U_)) = 1) // do they recover today and become susceptible today

095 inf_state[i] := S // move to state S

n_wt_strains[i] := n_hrp2_strains[i] := 0 // clear all strains

096 ENDIF

097 ENDIF

// assess if individual has cleared a strain naturally

// ----------------------------------------------------

098 IF (inf_state[i] = D OR A OR U OR T) // if individual is infected

099 total_strains := n_hrp2_strains[i] + n_wt_strains[i] // all strains present

// if they have more than 1 strain to clear AND will they clear a strain today

100 IF (total_strains > 1 AND bernoulli(1 - exp(-(total_strains)/(d_A_ + d_U_))) = 1)

// once chosen to clear a strain, which strain is chosen from relative ratio

101 IF (bernoulli(n_hrp2_strains[i]/total_strains)=1)

102 n_hrp2_strains[i] := n_hrp2_strains[i] – 1 // clear an hrp2-deleted strain

103 ELSE

104 n_wt_strains[i] := n_wt_strains[i] – 1 // clean a wild type strain

105 ENDIF

106 ENDIF

107 ENDIF

// assign non-normalised age dependent biting heterogeneity

108 REPEAT LINES 36-37

// assign contributions to onward infectiousness

109 REPEAT LINES 21-35

110 ENDFOR

// normalise age-dependent biting heterogeneity

111 REPEAT LINES 39-41

// ----------------------------------------------------

// END NON-INFECTION CHANGES

// BEGIN MOSQUITO DYNAMICS

// ----------------------------------------------------

// calculate strain profile and size of human infectious reservoir that is gametocytogenic today

112 hrp2_wt_reservoir := hrp2_del_reservoir := 0 // reset reservoir sums to zero

113 FOR i := 1 TO N

// what was the total number of strains that could now be gametocytogenic

114 total_gam_strains[i] := (n_hrp2_strains[i] + n_wt_strains[i] FROM delay_gam EARLIER)

// what is the relative contribution of hrp2 deleted strains to onward transmission today, which is determined by the contribution to onward infection from delay_gam days earlier

115 hrp2_del_reservoir := hrp2_del_reservoir + ($\psi$[i]*$\zeta$[i]*cont[i]*n_hrp2_strains[i]) FROM delay_gam EARLIER))/total_gam_strains[i]

// what is the relative contribution of wild type strains to onward transmission today, which is determined by the contribution to onward infection from delay_gam days earlier

116 wt_reservoir := wt_reservoir + ($\psi$[i]*$\zeta$[i]*cont[i]*n_wt_strains[i]) FROM delay_gam EARLIER))/total_gam_strains[i]

117 ENDFOR

// calculate total size of human infectious reservoir and update mosquito population

118 human_reservoir := hrp2_del_reservoir/N + wt_reservoir/N

// mosquito population changes

119 surv := exp(-$\mu$_M_*delay_mos) // the proportion of mosq that survive long enough to complete the latent period

120 foiv := a_k_*human_reservoir // force of infection to vectors from delay_gam earlier

121 ince := S_M_*foiv // proportion of susceptible mosquitoes that become infected

122 incv := ince*surv //proportion of mosquitoes that get infected that survive to have sporozoites

123 Mv := S_M_ + E_M_ + I_M_ // total mosquito population size

124 betav := M_v_*$\mu$_M_ // growth rate of new mosquitoes that ensures constant population size

125 S_M_ := S_M_ + (-ince - ($\mu$_M_*S_M_) + betav) // susceptible size – infections - death + birth

126 E_M_ := E_M_ + (ince - incv - ($\mu$_M_*E_M_)) // exposed size + infections – incubations - death

127 I_M_ := I_M_ + (incv - ($\mu$_M_*I_M_)) // infectious size + incubations - death

// ----------------------------------------------------

// END MOSQUITO DYNAMICS

// loop through population deciding if they are bitten today using the entomological innoculation rate from delay-liver days earlier, if so does it lead to an increase in IB or not, does it lead to an infection and do ICA and ID subsequently increase or not, what strains will be passed on if it led to an infection by looking at the strain profile of the human infectious reservoir from delay_liver + delay_mos before, what infection state they will move to as a result of immunity and their strain profile that is detectable today along with RDT nonadherence and alternative diagnosis using microscopy-based detection, before considering whether that person will have also potentially been treated due to non-malarial fever.

// BEGIN MOSQUITO BITING

// ----------------------------------------------------

128 FOR i := 1 TO N

// are they bitten by an infectious mosquito

129 IF (bernoulli(1-exp(-(a_k_*$\zeta$[i]*$\psi$[i]*(I_M_ FROM delay_liver EARLIER))) = 1)

// was their last boost time to pre-erythrocytic immunity more than u_B_ days earlier then boost immunity and set last boost time to today

130 IF ((t - t_k_[i]) > u_B_)

131 IB[i] := IB[i] + 1 // increase pre-erythrocytic immunity

132 t_k_[i] := t // set last boost time to today

133 ENDIF

// calculate the probability of being infected

134 b[i] := b_0_ * (b_1_ + ((1 - b_1_) /(1 + ((IB[i] /I_B0_)^k_B_))))

// are they capable of being infected, i.e. not being treated or in prophylaxis

135 IF (inf_state[i] = S OR D OR A OR U))

// are they infected

136 IF (bernoulli(b[i]) = 1)

// was their last boost time to acquired clinical immunity more than u_B_ days earlier then boost immunity and set last boost time to today

137 IF ((t - t_l_[i]) > u_C_)

138 ICA[i] := ICA[i] + 1 // increase acquired clinical immunity

139 t_l_[i] := t // set last boost time to today

140 ENDIF

// was their last boost time to detection immunity more than u_B_ days earlier then boost immunity and set last boost time to today

141 IF ((t - t_m_[i]) > u_D_)

142 ID[i] := ID[i] + 1 // increase detection immunity

143 t_m_[i] := t // set last boost time to today

144 ENDIF

// calculate probability of developing symptoms

145 $\phi$[i] := $\phi$_0_ * ($\phi$_1_ + ((1 -$\phi$_1_) /(1 + (((ICA[i]+ICM[i]) /I_C0_)^$\kappa$_C_))))

// work out what strain they will be passed on by conducting a bernoulli trial using the relative ratio of hrp2 deleted strains to wild type strains from 22 days earlier, and applying any assumed fitness cost to hrp2 deletion which will shift the ratio.

146 IF (bernoulli((hrp2_del_reservoir*fitness_cost)/human_reservoir FROM (delay_liver + delay_mos) EARLIER)=1)

147 n_hrp2_strains[i] := n_hrp2_strains[i] + 1 // increase hrp2 deleted strains

// if they are subpatent we need to handle the chance that a positive test result will happen due to HRP3 epitopes differently than other diseased states. This is because we assume that strains inherited earlier will not be detected due to the individual being subpatent. As such $\varepsilon$ will only become 1 if the strain to be passed on is wild type. This will be the same for a susceptible individual who has no current strains.

148 IF (inf_state[i] = S OR U)

149 $\varepsilon$[i] := 0.25 // 25% chance of HRP3 epitope cross reactivity

150 ELSE

151 IF (n_wt_strains > 0)

152 $\varepsilon$[i] := 1 // individual has at least one wt strain and thus $\varepsilon$ = 1

153 ELSE

154 $\varepsilon$[i] := 0.25 // 25% chance of HRP3 epitope cross reactivity

155 ENDIF

156 ENDIF

157 ELSE

158 n_wt_strains[i] := n_wt_strains[i] + 1

159 $\varepsilon$[i] := 1 // individual has at least one wt strain and thus $\varepsilon$ = 1

160 ENDIF

// state change assignment by working out probability of being correctly detected if seeking treatment. This takes into account strain profile, and chance that they will still be treated despite being only infected with hrp2 deleted strains due to HRP3 epitope effects, non-adherence to test results and whether microscopy based detection was used

161 prob_hrp2_del_treated := 1 - ((1-$\varepsilon$[i])*(1-non_adherence)*(1-microscopy_use))

162 prob_D := $\phi$[i]*(1-(f_T_*prob_hrp2_del_treated)) // probability going to state D

163 prob_T := $\phi$[i]*(f_T_*prob_hrp2_del_treated) // probability of going to state T

// we assume that if they are in state D already an additional infection cannot lead them to become asymptomatic and thus the probability of going to state A is 0 if in state D, otherwise determined by 1 – probability of developing symptoms

164 IF (inf_state[i] = D)

165 prob_A := 0 // probability going to state A is 0% for those in state D

166 ELSE

167 prob_A := 1 - $\phi$[i] // probability going to state A if not in state D

168 ENDIF

// assign what state change occurs by sampling according to the probabilities of each state change occurring

169 inf_state[i] := DRAW FROM [D,T,A] WITH WEIGHTS [prob_D,prob_T,probA]

170 ENDIF

171 ENDIF

172 ENDIF

// ----------------------------------------------------

// END MOSQUITO BITING

// BEGIN NON-MALARIAL FEVER CONSIDERATION

// ----------------------------------------------------

// did a non-malarial fever occur using the age-dependent rate of non-malarial fevers

173 IF (bernoulli(1-exp(-nmf[age[i]])) = 1)

// if they are susceptible the only chance of being treated is if they both seek treatment and the outcome of the test result is not adhered to. If so they will move to a state of prophylaxis rather than being treated as they have no strains to be treated

174 IF (inf_state[i] = S) // if in state S

175 IF (bernoulli(f_T_*non_adherence)) // if they seek treatment and result is not adhered to

176 inf_state[i] := P // move to state P

177 ENDIF

178 ENDIF

// if they are subpatent the only chance of being treated is if they both seek treatment and the outcome of the test result is not adhered to. If so they will move to being treated

179 IF (inf_state[i] = U) // if in state U

180 IF (bernoulli(f_T_*non_adherence)) // if they seek treatment and result is not adhered to

181 inf_state[i] := T // move to state T

182 ENDIF

183 ENDIF

// if they are diseased or asymptomatic they will be treated if they seek treatment and it either leads to a positive result if they have wild type strains or if they used microscopy for detection or the test result is not adhered to

184 IF (inf_state[i] = [D OR A]) // if in state D or A

185 IF (n_wt_strains > 0) // if they have any wild type strains

186 $\varepsilon$[i] := 1 // 100% probability of positive test result

187 ELSE

188 $\varepsilon$[i] := 0.25 // 25% chance of positive test result due to HRP3 epitope effects

188 ENDIF

// probability of being treated due to HRP3 epitope effects, or non-adherence to test result or microscopy based diagnosis used

189 prob_hrp2_del_treated := 1 - ((1-$\varepsilon$[i])*(1-non_adherence)*(1-microscopy_use))

190 IF (bernoulli(f_T_*prob_hrp2_del_treated)) // if seek treatment and are thus treated

191 inf_state[i] := T // move to state T

192 ENDIF

193 ENDIF

194 ENDIF

// ----------------------------------------------------

// END NON-MALARIAL FEVER CONSIDERATION

195 ENDFOR

196 ENDFOR

// ----------------------------------------------------

// END STEPPING THROUGH TIME

^1^ The equilibrium solution to an equivalent deterministic model can be sought by categorising the age and individual biting heterogeneity of individuals into a finite number of bands. The equations for the deterministic model are detailed below.

$$\frac{\partial S}{\partial t}+ \frac{\partial S}{\partial a}= -\Lambda S+\frac{P}{d_{P}}+\frac{U}{d_{U}}$$

$$\frac{\partial T}{\partial t}+ \frac{\partial T}{\partial a}= \phi f_{T}\varepsilon\Lambda(S+D+A+U)-\frac{T}{d_{T}}$$

$$\frac{\partial D}{\partial t}+ \frac{\partial D}{\partial a}= \phi{(1-f}_{T}\varepsilon)\Lambda(S+A+U)-\frac{D}{d_{D}}$$

$$\frac{\partial A}{\partial t}+ \frac{\partial A}{\partial a}=\left( 1-\phi\right)\Lambda\left( S+U \right)+\frac{D}{d_{D}}-\phi\Lambda A- \frac{A}{d_{A}}$$

$$\frac{\partial U}{\partial t}+ \frac{\partial U}{\partial a}= \frac{A}{d_{A}}-\frac{U}{d_{U}}-\Lambda U$$

$$\frac{\partial P}{\partial t}+ \frac{\partial P}{\partial a}= \frac{T}{d_{T}}- \frac{P}{d_{P}}$$
