# Supplementary material for: Modelling the drivers of the spread of Plasmodium falciparum hrp2 gene deletions in sub-Saharan Africa
Source: eLife. 2017 Aug 24;6:e25008. doi: 10.7554/eLife.25008 (PMC5602420; doi:10.7554/eLife.25008)
Supplement: Transparent reporting form [file elife-25008-transrepform.pdf]

eLife's transparent reporting form

We encourage authors to provide detailed information *within their submission* to facilitate the interpretation and replication of experiments. If you have any questions, please contact us: editorial@elifesciences.org.

Sample-size estimation

- You should state whether an appropriate sample size was computed when the study was being designed
- You should state the statistical method of sample size computation and any required assumptions
- If no explicit power analysis was used, you should describe how you decided what sample (replicate) size (number) to use

Please outline where this information can be found within the submission (e.g., page numbers or figure legends), or explain why this information doesn't apply to your submission:

The outputs in the paper are model-based and so standard statistical sample size calculations do not apply. However, we have highlighted here where we justify simulation assumptions.

For all simulations carried out, 10 simulation repeats were carried with substantial population sizes equal to 100,000 (page 20). These values were considered acceptable, yielding suitably small variation when examining gradual parameter sweeps, such as in in Figure 2 (page 5) and Figure 4 – supplement 2.

Replicates

- You should report how often each experiment was performed
- You should include a definition of biological versus technical replication
- The data obtained should be provided and sufficient information should be provided to indicate the number of independent biological and/or technical replicates
- If you encountered any outliers, you should describe how these were handled
- Criteria for exclusion/inclusion of data should be clearly stated
- High-throughput sequence data should be uploaded before submission, with a private link for reviewers provided (these are available from both GEO and ArrayExpress)

Please outline where this information can be found within the submission (e.g., page numbers or figure legends), or explain why this information doesn't apply to your submission:

For all simulations carried out, 10 simulation repeats were carried with substantial population sizes equal to 100,000 (page 20). These represent stochastic uncertainty, which at this population size is quite small. No simulations were rejected in analysis, with the population size being significantly large such that stochastic extinction did not occur.

Statistical reporting

- Statistical analysis methods should be described and justified
- Raw data should be presented in figures whenever informative to do so (typically when N per group is less than 10)
- For each experiment, you should identify the statistical tests used, exact values of N, definitions of center, methods of multiple test correction, and dispersion and precision measures (e.g., mean, median, SD, SEM, confidence intervals; and, for the major substantive results, a measure of effect size (e.g., Pearson's r, Cohen's d)
- Report exact p-values wherever possible alongside the summary statistics and 95% confidence intervals. These should be reported for all key questions and not only when the p-value is less than 0.05.

Please outline where this information can be found within the submission (e.g., page numbers or figure legends), or explain why this information doesn't apply to your submission:

The paper contains simulation modelling output and as such no formal statistical hypothesis tests were conducted. The model was calibrated to the DRC data using a least-squares approach (page 7 Figure 3, and page 20). As this is not a full formal fitting method, uncertainty intervals for the estimated parameter are not appropriate to report.

(For large datasets, or papers with a very large number of statistical tests, you may upload a single table file with tests, Ns, etc., with reference to page numbers in the manuscript.)

Additional data files ("source data")

- We encourage you to upload relevant additional data files, such as numerical data that are represented as a graph in a figure, or as a summary table
- Where provided, these should be in the most useful format, and they can be uploaded as "Source data" files linked to a main figure or table
- Include model definition files including the full list of parameters used
- Include code used for data analysis (e.g., R, MatLab)
- Avoid stating that data files are "available upon request"

Please indicate the figures or tables for which source data files have been provided:

Source data is provided for each figure, with suitably labelled csv files for each plot. The simulation code is available online (see Source Code section), with the scripts and source data for each figure plot also provided with the source code.
